# Electrically Pumped III-N Microcavity Light Emitters Incorporating an Oxide Confinement Aperture

**DOI:** 10.1186/s11671-016-1801-2

**Published:** 2017-01-05

**Authors:** Ying-Yu Lai, Tsu-Chi Chang, Ya-Chen Li, Tien-Chang Lu, Shing-Chung Wang

**Affiliations:** Department of Photonics, National Chiao Tung University, 1001 University Road, Hsinchu, 300 Taiwan

**Keywords:** Microcavity, Light emitting diodes (LEDs), Electrically pumped, Oxide aperture

## Abstract

In this work, we report on electrically pumped III-N microcavity (MC) light emitters incorporating oxide confinement apertures. The utilized SiO_2_ aperture can provide a planar ITO design with a higher index contrast (~1) over other previously reported approaches. The fabricated MC light emitter with a 15-μm-aperture shows a turn-on voltage of 3.3 V, which is comparable to conventional light emitting diodes (LEDs), showing a good electrical property of the proposed structure. A uniform light output profile within the emission aperture suggesting the good capability of current spreading and current confinement of ITO and SiO_2_ aperture, respectively. Although the quality factor (*Q*) of fabricated MC is not high enough to achieve lasing action (~500), a superlinear emission can still be reached under a high current injection density (2.83 kA/cm^2^) at 77 K through the exciton-exciton scattering, indicating the high potential of this structure for realizing excitonic vertical-cavity surface-emitting laser (VCSEL) action or even polariton laser after fabrication optimization.

## Background

III-Nitride based materials are highly attractive for making vertical microcavity (MC) light emitters such as strongly coupled polariton lasers and conventional vertical-cavity surface-emitting lasers (VCSELs) due to their large exciton binding energies and wide spectra tuning range in the ultraviolet-visible region [1–12]. So far, electrically pumped polariton emitters and conventional VCSELs have been demonstrated in III-nitride based MCs at room temperature (RT). However, the p-side designs including indium-tin-oxide (ITO) transparent conducting layer (TCL) and dielectric materials based current aperture in the reported devices induce a negative guiding effect and lateral optical loss [13–15]. To provide a sufficient positive guiding effect, Cheng et al. utilized an intra-cavity low-index AlN layer to serve as a lateral optical/current aperture and achieved a high-quality-factor (*Q*) emission; however, no lasing action was observed in their MC light emitter due to the lack of TCL [16]. A similar work, in which an oxidized AlInN aperture has been implanted in to a GaN-based LED, has been demonstrated by Castiglia et al. [17]. In 2014, a GaN-based MC emitter featuring a Si-diffusion-defined current aperture developed by Yeh et al. shows a good current confinement capability [18]. Although the current flow can be well-confined in the non-Si-diffused area, the near zero index contrast between diffused and non-diffused area cannot provide a sufficient lateral optical confinement. Recently, Leonard et al. proposed an approach using ion-implantation to define the current aperture. In their result, the index contrast between the implanted and the non-implanted p^++^GaN can be enhanced to 0.05 [9]. In this letter, we propose an alternative design using self-aligned SiO_2_ aperture featuring a low index and high resistance. Within this structure, the index contrast between the p-GaN and surrounded SiO_2_ aperture can be enlarged to about 1 and provide a better lateral optical confinement. Through this approach, a multi-transverse mode behavior resulted from the strong lateral optical confinement can be observed in the measured spectra. Moreover, a strong excitonic gain emission generated from the devices with a small aperture size reveals the possibility of such devices to be pushed into lasing action or even polaritonic operation [19, 20].

## Methods

**Fig. 1:**
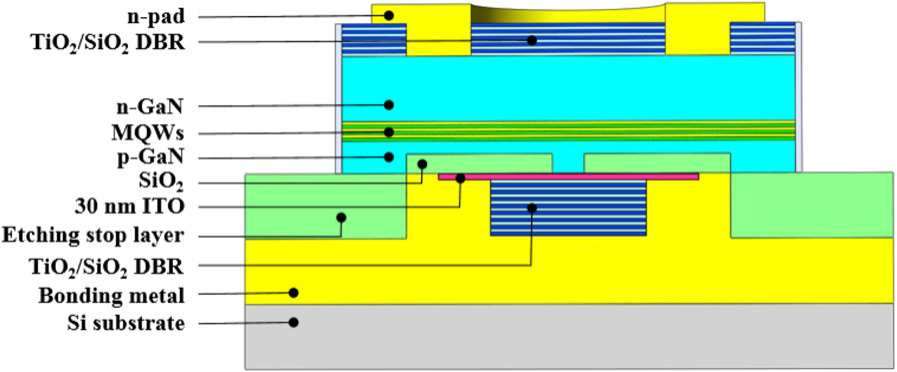
Schematic of the proposed oxide-confined electrically pumped III-N MC structure

**Fig. 2:**
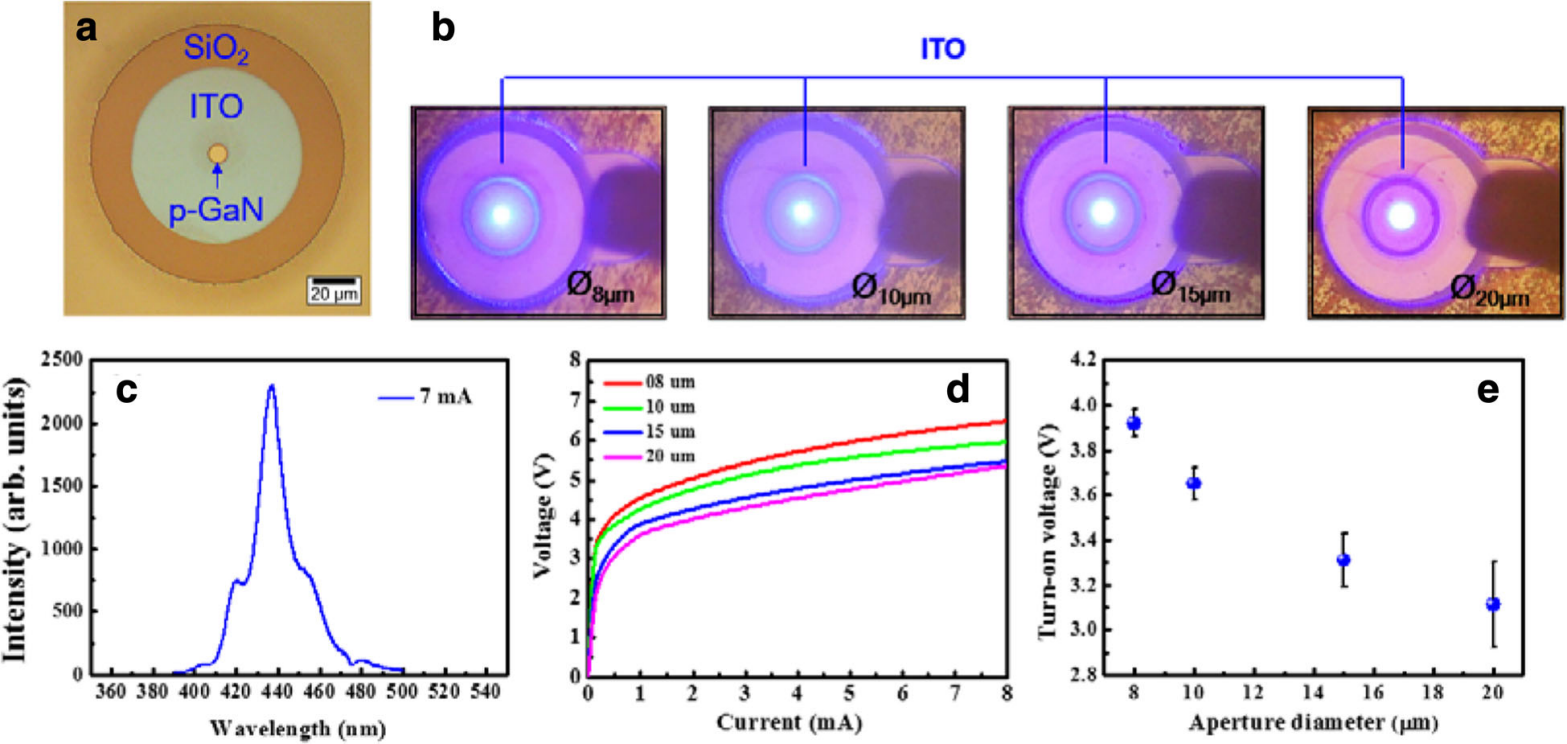
**a** Top view of device after ITO deposition. **b** Near-field images of half-cavity devices with various apertures under 7 mA injection current at RT. **c** Emission spectrum of a half-cavity device with a 15-μm-diameter oxide aperture. **d** Voltage-current characteristics of whole-cavity device with different aperture sizes at RT. **e** Extracted turn-on voltages of whole-cavity device as a function of diameter of oxide aperture

The schematic diagram of the proposed electrically pumped III-N MC featuring a planar low-index SiO_2_ aperture is shown in Fig. 1. The III-N cavity layers were grown on a c-plane sapphire substrate by metal-organic chemical vapor deposition (MOCVD), followed by a thin GaN nucleation layer, a 2-μm undoped GaN (u-GaN) spacer, and a *p-i-n* diode structure. The diode structure consisted a 2-μm n-GaN layer (doping level 4 × 10^18^ cm^−3^), ten pairs of In_0.2_Ga_0.8_N/GaN (2.5 nm/12.5 nm) multiple quantum wells, and a 150-nm p-GaN layer (doping level 4 × 10^19^ cm^−3^). After epitaxial growth, a 90-nm height p-GaN mesa was formed by a ring-shaped photoresist layer and inductively coupled plasma reactive ion etching (ICP-RIE) etching process. Before removing the photoresist, a 90-nm-thick SiO_2_ layer was deposited by an E-gun coater to act as the low-index planar current aperture. A 30-nm-thick ITO TCL featuring a surface roughness of 1 nm was then deposited by a reactive plasma deposition system, as shown in Fig. 2a. Then, a circular patterned 12-pair TiO_2_/SiO_2_ DBR was deposited on top of the ITO layer using an in situ optically monitored E-gun deposition system. The measured reflectivity exceeds 99% due to the high index contrast between TiO_2_ and SiO_2_. Next, a ring-shape p-contact consisting Cr/Pt/Au alloy was deposited on the ITO layer. The sample was then bonded onto an In/Au capped Si wafer for the following laser lift-off (LLO) process [21]. After LLO, the rough u-GaN layer was removed by the polishing process. To achieve a shorter cavity length, the residue n-GaN layer was thinned and polished to about 1.5 μm by ICP-RIE and mechanical polishing process. The surface roughness (1.1 nm) of the exposed n-GaN layer is measured by atomic force microscopy with a 5 × 5 μm^2^ scanned area. After device isolation, an electroluminescence (EL) measurement was performed at RT under 7 mA continuous wave (CW) current injection, and the corresponding emission image from n-GaN was collected by a CCD, as shown in Fig. 2b. From Fig. 2b, the luminescence light spots are confined inside the oxide apertures with different aperture size showing a good current confinement capability of proposed oxide-confined structure. After checking the current confinement, the emission was collected by a multimode fiber and transmitted into a spectrometer with a spectral resolution of about 0.2 nm. The Fabry-Perot (FP) resonance behavior between p-side DBR and n-GaN/air interface can be observed in Fig. 2c. The poor *Q* values of measured FP peaks are due to the low reflectance at n-GaN/air interface. After coating a TiO_2_/SiO_2_ n-side DBR, the sample was finished by the n-contact deposition. Figure 2d, e shows the measured voltage-current characteristics and extracted values of turn-on voltage of the fabricated MC light emitters with different aperture sizes. The turn-on voltage values are similar to that of the conventional LED, showing a favorable quality of sample fabrication. From Fig. 2e, device with larger aperture size has a lower turn-on voltage because of the shorter current path length between n-contact and p-contact. It is worth mentioning that the turn-on voltage of this work is much lower than that of previously reported AlN-buried MC emitter (~7 V) due to the good current spreading capability of ITO and the lower deposition temperature of SiO_2_ aperture comparing with the growing temperature of AlN, which would not cause unwanted damage to the InGaN MQW [16].

## Results and Discussion

**Fig. 3:**
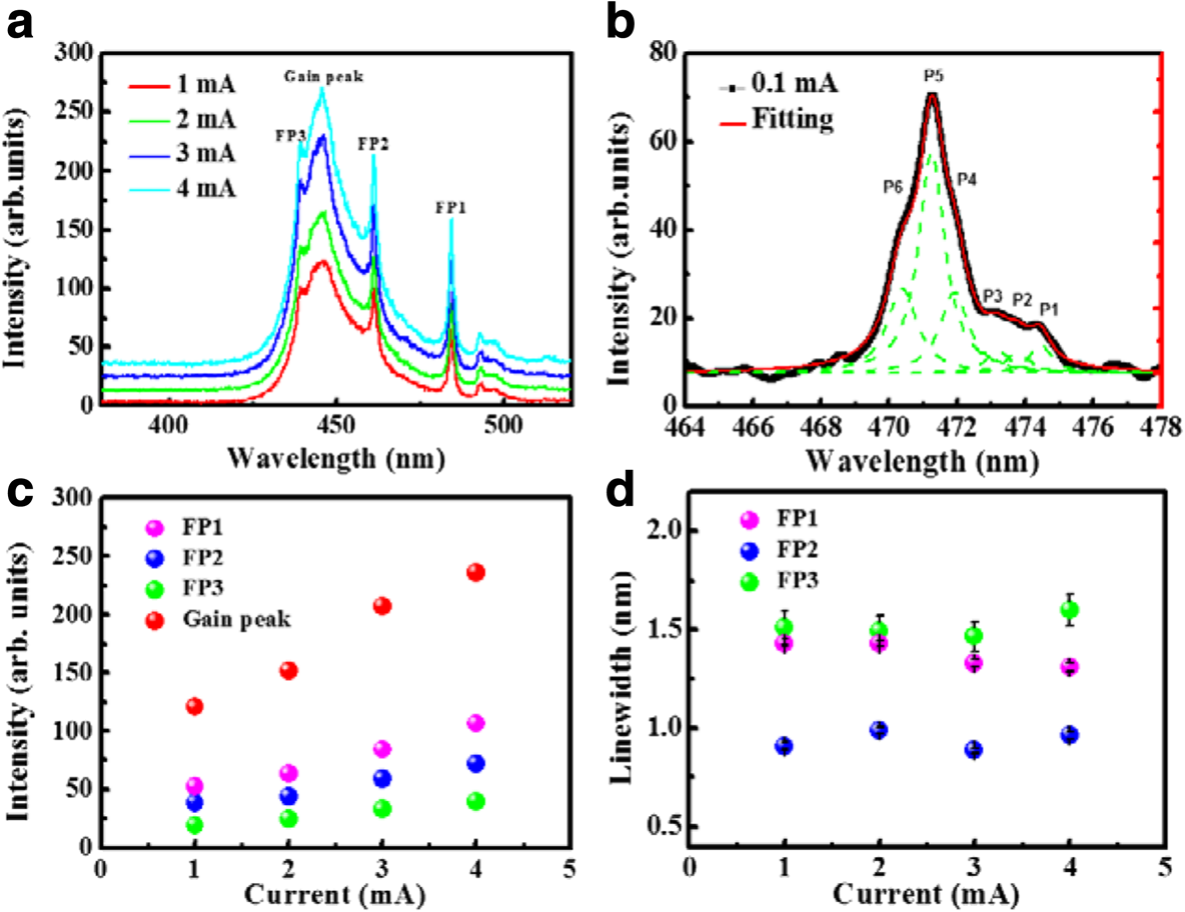
**a** RT spectra of a 15-μm-aperture MC device under different injection currents. **b** RT emission spectrum of a 15-μm-aperture MC device operated at 0.1 μA current injection. *Green dashed lines* represent the fitting curves of each transverse optical mode, and *red dashed lines* depict the summarized fitting curves. **c** Emission intensities and **d** linewidths of gain peak and FP modes extracted from the measured RT spectra as a function of injection current

Figure 3a shows the RT cavity emission spectra measured from a whole-cavity device with a 15-μm aperture diameter. To avoid the heating issue of CW injection scheme, the driving source operates at a pulse mode with a pulse width of 100 μs and a duty cycle of 5%. A MQW emitted gain peak and three FP modes observed in the measured spectra reflect the formation of MC. The cavity length calculated from the mode spacing between FP modes is about 1.9 μm. For a much lower injection condition (0.1 mA), FP1 shows a multi-peak behavior which corresponds to the transverse modes (P_1_ to P_6_) generated from the index-guiding effect of oxide aperture, as shown in Fig. 3b. The spatial mode size calculated from the measured mode spacing (0.7 nm) is about 1 μm, which is consistent with that of the result of Cheng et al. [16]. In Fig. 3c, the intensities of gain peak and FP modes show a linear increasing feature as a function of injection current, which is similar to conventional LED devices. Also the linewidth of the FP peaks in Fig. 3d shows no narrowing effect, implying that there has been no lasing action achieved. The *Q* value calculated from the linewidth is about 500. The reason why no lasing action was observed could be attributed to that the spatial mode overlapping between gain and FP mode in the longitudinal direction is not high enough to compensate the total loss of MC since the cavity thickness is difficult to be perfectly controlled during the polishing process.

**Fig. 4:**
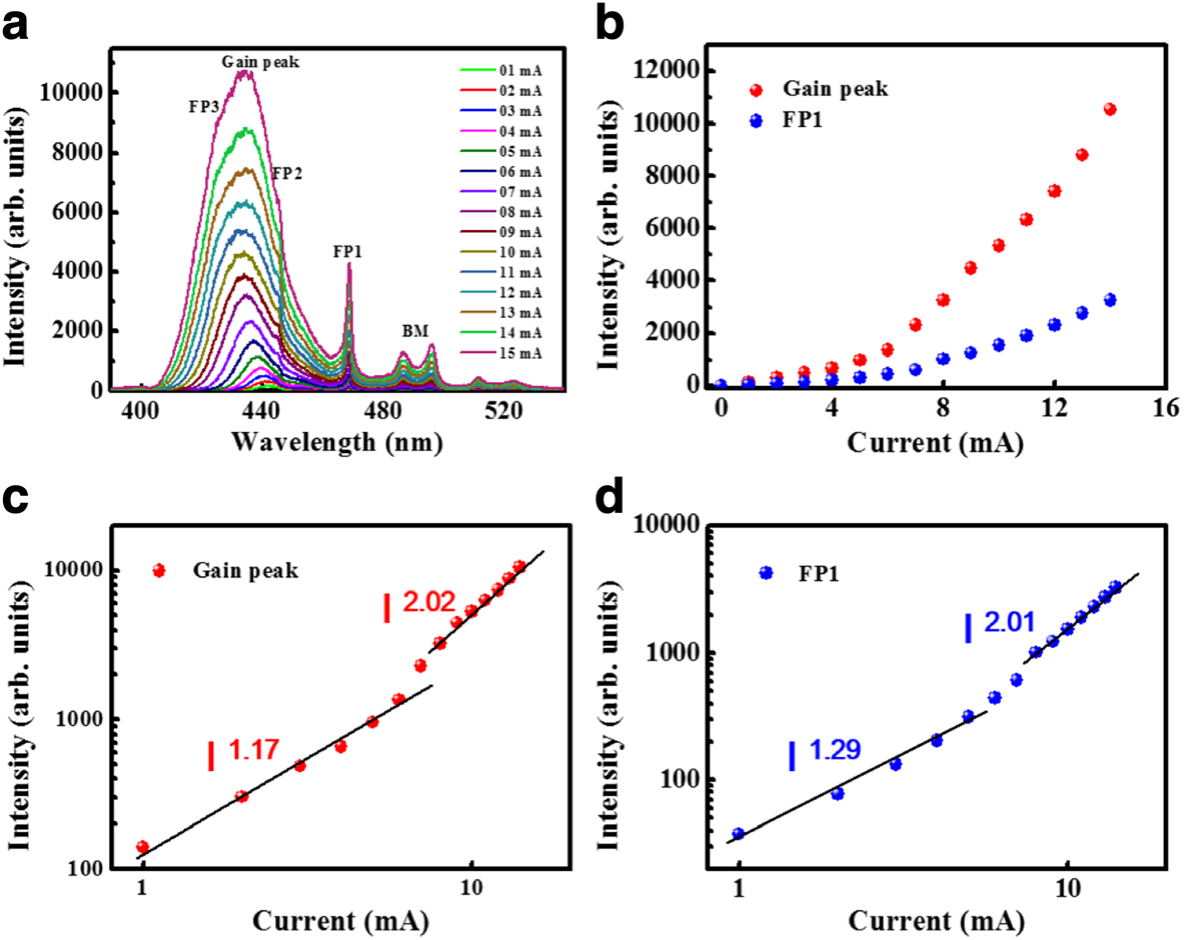
**a** Emission spectra of a 15-μm-aperture MC device under different injection currents at 77 K. **b** Measured emission intensities of gain peak and FP1 at 77 K as a function of injection current. Emission intensities of (**c**) gain peak and (**d**) FP1 in log scale vs injection current (linear scale) at 77 K. *Black fitting curves* correspond to the intensity variation vs injection current

To enhance the material gain for achieving lasing action, the sample was mounted inside a cryogenic chamber in which probe and multimode fiber are packaged. Using the liquid nitrogen circuit, we can perform the EL measurement at 77 K. From the EL spectra shown in Fig. 4a, both of the gain peak and the FP modes are still observable. Due to the higher material gain at 77 K compared with the RT case, some of FP modes such as FP2 and FP3 are buried in the strong gain peak and are not clearly observable. The Bragg mode (BM) peaks located around 500 nm are originated from the optical leakage at the stopband edge of DBR. Figure 4b shows the extracted peak intensities of gain mode and FP1 as a function of driving current, both of them show a superlinearly increasing phenomena. In the log-log scale intensity variation shown in Figs. 4c, d, the slopes of gain and FP modes jump from around 1 to 2 as the injection current reaching 5 mA (corresponds to 2.83 kA/cm^2^ current density). There was no linewidth narrowing effect observed from the spectra, indicating that lasing action is still not achieved at 77 K. This superlinear emission (power factor of 2) and clear blue-shift characteristics should be related to the onset of exciton-exciton (X-X) scattering, which is usually observed in wide bandgap materials [20, 21]. The wide bandwidth of X-X scattering emission can be attributed to the multi-localized-mode emission of InGaN MQW, in which indium atoms are localized and inhomogeneously distributed. Although material gain was enhanced at 77 K, the un-compensable optical loss that induced by poor longitudinal mode overlapping and low *Q* value limit the fabricated MC to experience only the X-X scattering instead of lasing action at a high pumping condition. This also explains why the measured intensities of FP1 mode shows a power factor with the gain band with no linewidth narrowing since it comes from the filtered result of the broadband X-X scattering.

## Conclusions

In summary, we have demonstrated an III-N MC emitter featuring an oxide confinement aperture which can be driven by electrical injection. Thanks to the large index contrast between oxide and III-N (~1), our design can provide a better optical confinement than other reported fabrication techniques such as ion-implantation and Si-diffusion which index contrasts are about or even smaller than 0.1. From the CCD images and EL measurement results, oxide confinement aperture can provide a uniform light emission within the aperture but also maintain good electrical properties which are comparable with conventional LED devices. Also, a multi-transverse mode characteristic was observed under low injection condition. Interestingly, under high injection at 77 K, a pronounce X-X scattering with superlinearly increased intensity can be achieved in the fabricated MC devices with a 2.83 kA/cm^2^ threshold current density. Our demonstration suggests a powerful design for the electrically pumped III-N MC with excitonic gain which could be applied to low threshold MC laser devices including VCSELs and polariton lasers.
